# A fungal effector hijacks a plastid protein to dampen plant immunity; PR1 is here for rescue

**DOI:** 10.1007/s44154-025-00230-z

**Published:** 2025-04-02

**Authors:** Muhammad Saad Shoaib Khan, Faisal Islam, Huan Chen, Jian Chen

**Affiliations:** 1 International Genome Center, Jiangsu University, Zhenjiang, 212013 China; 2 Joint Center of Single Cell Biology, School of Agriculture and Biology, Shanghai Jiao Tong University, 800 Dongchuan Road, Shanghai, 200240 China

## Abstract

Plants are engaged in a constant battle for survival against pathogens, which triggers a multifaceted immune response characterized by pattern-triggered immunity (PTI) and effector-triggered immunity (ETI) to prevent infection. These two immune responses operate synergistically to enhance plant immunity. PTI is considered the first line of defense involving the recognition of pathogen-associated molecular patterns (PAMPs) by specific receptors in host cells known as pattern recognition receptors (PRRs), which initiate defense signaling. However, many pathogens often overcome the first line of defense (PTI) and successfully deploy effector proteins to promote virulence and subvert plant immunity, leading to host susceptibility. In the counter-defense, the ETI defense mechanism is activated by triggering resistance (R) genes in plants that usually encode nucleotide-binding-leucine-rich-containing (NLR) proteins. During plant-pathogen interactions, transcriptional reprogramming of defense-related genes such as pathogenesis-related proteins and generation of reactive oxygen species (ROS) are essential for facilitating programmed cell death at the infected location to inhibit pathogen proliferation. While ROS and PR protein are critical in plant-pathogen interaction, they are not universally required or effective against all pathogens. Hence, plants’ multilayer immune layer is encrypted with the compensatory activation of ETI defense response towards the failure of one component of the defense system to maintain robust immunity.

## Plant PR1 and ISP: The immunity drivers against pathogenic effectors

In plants' immune signaling, pathogenesis-related protein (PR1) is recognized as a marker of salicylic acid (SA)-mediated defenses. *PR1* gene is involved in the generation of small signaling peptides known as cysteine-rich secretory protein, antigen5, and Pathogenesis-related 1 (CAP). The CAP-derived peptide 1/9 (CAPE1/9) serves as elicitor to initiate immunity in the host cell (Chen et al. 2014; Chen et al. 2023). The CAPE1 protein is also found in pathogens like fungus, and deletion of this protein leads to compromised virulence, indicating the dual defensive and offensive characteristics of CAP proteins (Han et al. 2023; Li et al. 2023). Previous research demonstrated that during a pathogen attack, triggers SA-mediated immune response and PR1 is secreted into the apoplast, where the CAP domain of the PR1 protein binds with the sterol of the fungal membrane and causes its sequestration, thereby compromising the virulence of the pathogen (Breen et al. 2017; Gamir et al. 2017). To date, five different pathogen effectors have been identified to target PR1 protein and inhibit its antimicrobial activity. Among these effector proteins, a novel cysteine-rich protein known as Cerato-plantain (CP), comprising of 105–134 amino acid CP domain has recently caught the attention of the scientific community. Additionally, several fungal pathogens like *Botrytis cinerea*, *Magnaporthe oryzae*, *Fusarium oxysporum* f. sp. *Lycopersici* (*Fol*), etc., have been shown to rely on CP-comprising effectors (CPE) to facilitate pathogenicity (Sung et al. 2021).

**Fig. 1:**
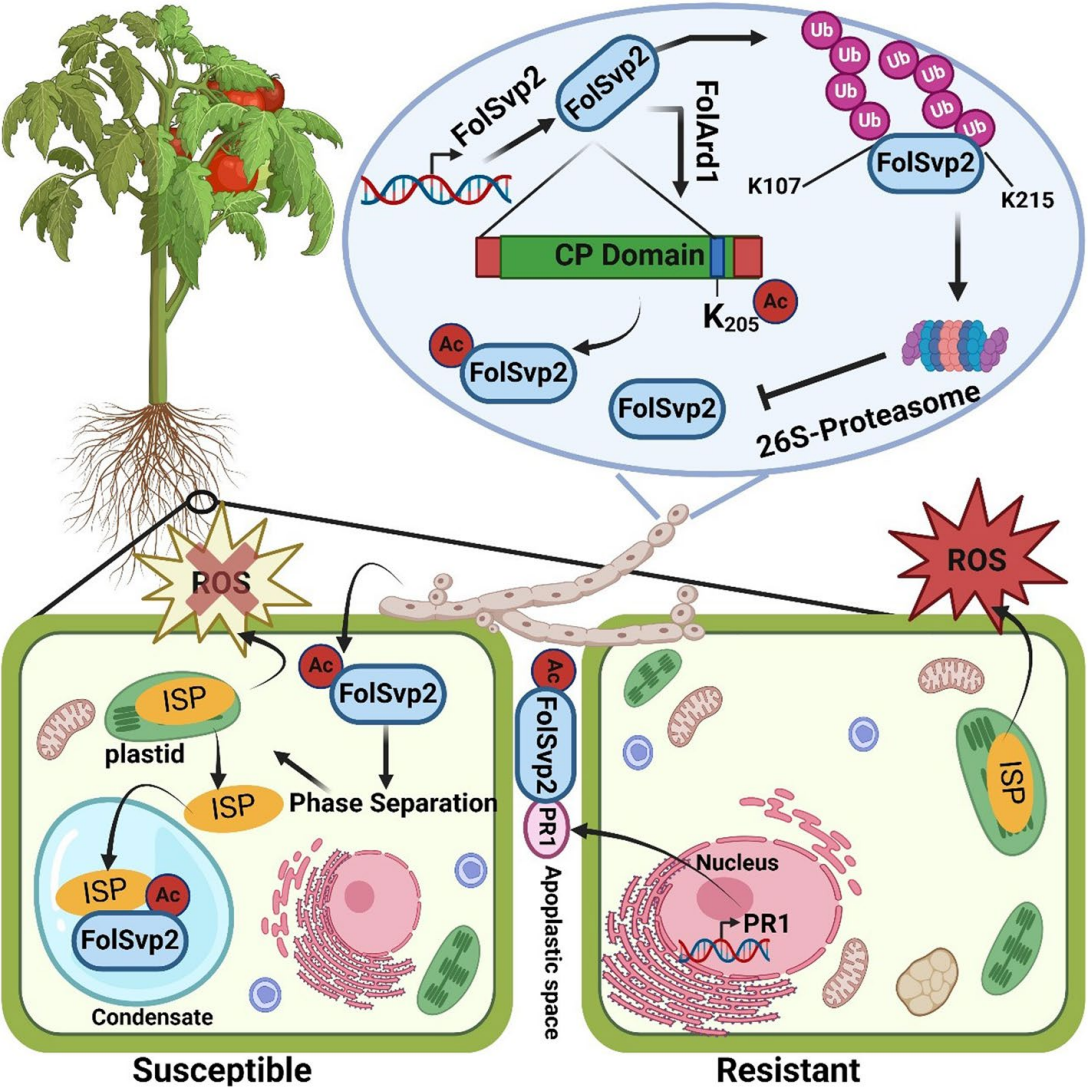
A hypothetical model representing the interplay between the PR1 of the host plant and FolSvp2 of *Fusarium oxysporium* f. sp. *Lycopersici* (*Fol*). Inside the fungal hype, the ubiquitination of the FolSvp2 effector at two distinct K107 and K215 residues leads to degradation by 26S proteasome. Acyltransferase FolArd1 recuses the FolSvp2 from degradation and acetylates K205 of FolSvp2. The acetylated FolSvp2 enters the host cell and translocates the SlISP from the plastid to the effector condensate phase separation. As a result, the SlISP could not send signals to generate ROS and consequently leading to susceptibility in plants. After the hijacking of SlISP, the plant defense system launches a rescue operation, by activating the disease-resistant gene *PR1* in the nucleus, which is translated and blocks the entry of the FolSvp2 effector at the apoplastic space and mitigates fungal infection

Iron-sulfur proteins (ISPs) are a group of 40 different proteins residing in plastids and responsible for photo-respiration, physiological and defense-related activities. ISP contributes to basal and induced resistance in plant cells through the modulation of the chloroplast-derived redox signaling network. The plant immunity is suppressed when ISP is targeted by pathogen effectors. For example, the *Meloidogyne javanica* nematode effector ROS suppressor (Mj-NEROSs) binds with ISP to alter the electron transport chain and reduce ROS production to facilitate pathogen proliferation. The SA-mediated defense gene like *PR1* has been reported to be indirectly regulated by ISP. The research stated that the silenced *atnfs* (Nitrogen fixation S like1; a member of ISP) mutants in Arabidopsis showed reduced expression of *PR1* gene and also increased susceptibility to *Pseudomonas syringae* pv. *tomato* DC3000 (Fonseca et al. 2020). Conversely, overexpression of *AtNFS1* leads to an eightfold increase in the expression of the *PR1* gene and enhanced disease resistance, which contributes to elevated expression of SA-mediated defense signaling genes. Hence, there is a positive interaction between ISP and SA-mediated *PR1* gene to confer plant immunity (Fig. 1).

## FolSvp2 drives phase separation to hijack ISP; PR1 is here to rescue

The plant cellular environment under siege by pathogens triggers ROS production, generally at chloroplast, mitochondria, apoplast, and peroxisomes (Stojilković et al. 2024). Several molecules participating in ROS generation are the prime targets for effectors in the ongoing struggle of pathogens to counteract plant immunity. Li et al. (2024) recently found a novel CPE named *Fusarium oxysporum* f. sp. *Lycopersici* (*Fol*) secreted Virulence-related Protein 2 (FolSvp2) in tomato plants infected with *Fol*. The secreted toxic proteins, FolSvp2, can enter the host cell and form membrane-less compartments through phase separation. Meanwhile, to counter the Iron-sulfur protein SlISP-triggered ROS defense system in tomatoes, the FolSvp2 manipulates the SlISP, hijacking it from plastids and co-aggregates with it. As a result of SlISP translocation, the ROS production attenuated in the host cell, consequently, increasing the pathogen infection. Further research revealed that acetylation modification of FolSvp2 is positively regulating the pathogenicity. The pathogen's acetyltransferase FolArd1 acetylates FolSvp2 at the lysine residue K205, and the acetylation at this site inhibits the ubiquitin-mediated degradation of FolSvp2 in both the fungus and the host, thus promoting the stability and toxicity of FolSvp2 (Li et al. 2024).

In plant immunity, when one defense component is compromised, another component is activated to take part in the defense response against pathogens; this phenomenon is called the compensatory defense mechanism (Tsuda and Katagiri 2010). The plant PR1 is one of the earliest discovered plant defense-related proteins. In response to pathogen infection, the host cells encounter the pathogen effectors by triggering the immunity driver, PR1 protein. The study found that to overcome the toxicity mediated by the effector protein FolSvp2, the tomato PR1 protein interacts with FolSvp2 through its N-terminus in the extracellular space, thereby inhibiting the intracellular accumulation of the effector protein FolSvp2, restoring the plastid localization of the host plant iron-sulfur protein SlISP, and maintaining the tomato's resistance to the pathogen. Hence, PR1 inhibits the FolSvp2 from hijacking the ISP and mitigates the fungal infection. Thus, the mechanism by which N-terminal PR1 physically interacts with pathogen effector differs from its antifungal activity and its role in CAPE-triggering immune response.

## Concluding remarks and future prospects

The work by Li et al*.* (2024) provides a significant interplay of three proteins, FolSvp2, the oppressor; SlISP, the victim; and PR1, the rescuer. These three proteins contribute a major role in the regulation of pathogenicity and plant defenses respectively, in the host cell. The research results highlight the mechanism of a novel effector FolSvp2, which utilizes the host cellular environment through phase separation and translocates a plastid immunity component SlISP into effector condensates, resulting in the inhibition of ROS production and promoting the pathogen infection. The primary task of SlISP in plastid is regulating the photosynthetic electron transport chain. Recently, its involvement in plant immunity was also discovered, but its direct/indirect involvement in the transportation of metal ions is uncertain; as some members of the ISP, like Aconitase and Cysteine Desulfurase (NFS1), are indirectly involved in iron and sulfur homeostasis, respectively (Balk and Pilon 2011). Further study is needed to analyze the pleiotropic effect of SlISP, which might facilitate cellular nutrient transport to win a potential tug-of-war for nutrients between the pathogens and the host cell. A recent study suggests that plant immunity mechanism promotes the withdrawal of nutrients from the infected tissue to prevent pathogens from acquiring the nutrients. For instance, during bacterial infection in Arabidopsis, the Sugar transporter 13 (STP13) is activated, and it translocates the sugar from apoplast to intercellular vacuoles resulting in a low food supply for bacterial proliferation (Yamada et al. 2016). The role of PR1 in defense mechanism has been extensively studied (Han et al. 2023; Sung et al. 2021), however, its interaction with phase separation-mediated pathogenic attack remains elusive. Similarly, the mechanism through which pathogenic effectors engage in phase separation to manipulate plant defenses is also unclear and requires in-depth knowledge of phase separation-mediated plant-pathogen interaction. This can be done by investigating intrinsically disordered regions (IDRs), the drivers of phase separation, to promote resistance in plants. Understanding the complex nature of IDRs and PR proteins interaction could open a new gateway for improving crop resistance against fungal pathogens, thereby ensuring food security in an era of escalating environmental challenges.

## Data Availability

Not applicable.

## Abbreviations

AtNFS1: Arabidopsis Nitrogen fixation S like1
ETI: Effector-triggered immunity
E3 ligases: E3 ubiquitin ligase
CAPE1: CAP-derived peptide 1
FolSvp2: *Fusarium oxysporum* F. sp. *Lycopersici* (*Fol*) secreted virulence-related protein 2
IDRs: Intrinsically disordered regions ISP, Iron-sulfur protein
NLR: Nucleotide-binding-leucine-rich-containing proteins
PAMPs: Pathogen-associated molecular patterns
PR1: Pathogenesis-related protein 1
PTI: Pathogen-associated molecular pattern-triggered immunity
R: Resistance
ROS: Reactive oxygen species
SA: Salicylic acid
